# Prognostic performance of the WIfI classification in diabetic patients with infrapopliteal CLTI after endovascular revascularization: a subgroup analysis of the PRIME-WIFI registry

**DOI:** 10.3389/fcvm.2026.1984270

**Published:** 2026-10-29

**Authors:** Henan Zheng, Julong Guo, Fan Zhang, Zhu Tong, Zibo Feng, Ziheng Wu, Meng Ye, Chunshui He, Qiang Li, Xin Fang, Lianrui Guo

**Affiliations:** 1 Department of Vascular Surgery, Xuanwu Hospital Capital Medical University, Beijing, China; 2 Department of Vascular Surgery, Liyuan Hospital Affiliated Tongji Medical College of Huazhong University of Science & Technology, Wuhan, China; 3 Department of Vascular Surgery, the First Affiliated Hospital, Zhejiang University School of Medicine, Hangzhou, China; 4 Department of Vascular Surgery, Renji Hospital, School of Medicine, Shanghai Jiaotong University, Shanghai, China; 5 Department of Vascular Surgery, Hospital of Chengdu University of Traditional Chinese Medicine, Chengdu, China; 6 Department of Vascular Surgery, The Affiliated Qingdao Hiser Hospital of Qingdao University, Qingdao, China; 7 Department of Vascular Surgery, Affiliated Hangzhou First People’s Hospital, School of Medicine, Westlake University, Hangzhou, China

**Keywords:** chronic limb-threatening ischemia, diabetes mellitus, endovascular revascularization, infrapopliteal disease, WIfI classification

## Abstract

**Introduction:**

The Wound, Ischemia, and foot Infection (WIfI) classification is widely used in chronic limb-threatening ischemia (CLTI). However, its prognostic performance in diabetic patients after endovascular revascularization remains unclear. We evaluated its prognostic and discriminatory performance in this population.

**Methods:**

This subgroup analysis of the prospective, multicenter PRIME-WIFI registry included diabetic patients with infrapopliteal CLTI undergoing endovascular revascularization. Associations between baseline WIfI stage and MAE (all-cause mortality, major amputation, or clinically driven target-limb reintervention), its individual components, amputation-free survival, clinical improvement, and 12-month VascuQoL change were assessed using Kaplan–Meier, multivariable Cox, and Harrell's C-index analyses.

**Results:**

A total of 707 diabetic patients (730 limbs) were analyzed; in the full PRIME-WIFI cohort, diabetes status showed a borderline interaction with WIfI stage for MAE (*P* for interactio*n* = 0.083). One-year freedom from MAE decreased across WIfI stages (89.5%, 77.4%, 78.0%, and 70.1% for stages 1–4; log-rank *P* = 0.004), and WIfI stage independently predicted MAE (HR: 1.31; 95% CI: 1.09–1.56; *P* = 0.0031). One-year AFS was 94.7%, 83.3%, 83.3%, and 74.2% (log-rank *P* = 0.0003), with higher WIfI stage associated with worse AFS (HR: 1.53; 95% CI: 1.24–1.89; *P* < 0.001). Overall survival was 94.7%, 87.8%, 86.1%, and 80.3% (log-rank *P* = 0.009), and WIfI stage was independently associated with mortality (HR: 1.52; 95% CI: 1.20–1.94; *P* < 0.001). Freedom from major amputation was 100.0%, 95.3%, 96.8%, and 92.3% (log-rank *P* = 0.02), with higher WIfI stage independently associated with major amputation (HR: 1.62; 95% CI: 1.07–2.57; *P* = 0.020). In contrast, freedom from CD-TLR did not differ significantly across stages (log-rank *P* = 0.60; HR: 0.92; 95% CI: 0.68–1.23; *P* = 0.564). Clinical improvement tended to decrease with increasing WIfI stage but did not reach statistical significance (*P* = 0.053). VascuQoL scores improved from baseline to 12 months across all WIfI stages, with no significant difference in the magnitude of improvement among stages (time-by-WIfI interaction *P* = 0.247).

**Conclusions:**

In this diabetic subgroup, WIfI retained prognostic value for MAE, AFS, mortality, and major amputation. However, discrimination was clearest between stages 1 and 4, with limited separation between stages 2 and 3. These findings support WIfI for broad risk stratification while highlighting the need for complementary assessment in diabetic CLTI.

## 1 Introduction

Chronic limb-threatening ischemia (CLTI) represents the terminal stage of peripheral artery disease (PAD), characterized by chronic ischemic rest pain, non-healing ulcers, or gangrene resulting from severe arterial insufficiency (1). Prognosis at this stage is poor, with reported 1-year major amputation and mortality rates each exceeding 20% (2).

Accurate risk stratification is critical for prognostication and clinical decision-making in CLTI. Traditional classification systems, such as Fontaine and Rutherford, focus primarily on ischemic symptoms but do not adequately incorporate the severity of tissue loss or infection until the most advanced stages. To address these limitations, the Society for Vascular Surgery (SVS) introduced the Wound, Ischemia, and foot Infection (WIfI) classification system in 2014. WIfI stratifies limb threat based on three domains—wound extent, severity of ischemia, and foot infection status—and aggregates them into four clinical stages reflecting increasing risk of major amputation within one year under medical therapy, ranging from stage 1 (very low risk) to stage 4 (high risk) (3). This framework offers a standardized and quantifiable approach to assessing limb threat and guiding revascularization decisions. Despite some concerns regarding interobserver variability and subgroup applicability, WIfI has been widely adopted and incorporated into international guidelines for diabetic foot ulcers (DFU) and CLTI (1, 4).

Among risk factors for CLTI, diabetes mellitus is both the most prevalent and the most potent. Present in over 50% of CLTI cases, diabetes is associated with significantly higher risks of limb loss and adverse clinical outcomes compared to non-diabetic individuals (5–8). These risks reflect distinct pathophysiologic and anatomic features of diabetic limb disease, including combined macrovascular and microvascular involvement, severe medial arterial calcification, endothelial dysfunction, peripheral neuropathy, and impaired immune response (9–11).

Within this context, diabetes may introduce additional complexity to the interpretation and performance of the WIfI classification. The three scoring domains—wound, ischemia, and foot infection—may interact in more variable ways in diabetic limbs. In particular, ischemia grading based on the ankle-brachial index (ABI) can be compromised by non-compressible, calcified vessels, while wound progression and infection severity may follow less predictable clinical trajectories. As a result, whether diabetes modifies the association between WIfI stage and clinical outcomes, and how well WIfI discriminates risk specifically within diabetic patients after revascularization, remain clinically relevant questions (3, 12, 13). Therefore, we conducted a subgroup analysis of the prospective, multicenter PRIME-WIFI registry to evaluate the prognostic performance and discriminatory ability of WIfI staging in diabetic patients with infrapopliteal CLTI undergoing endovascular therapy.

## 2 Materials and methods

### 2.1 Study design and population

This study was a sub-analysis of the PRIME-WIFI registry (ClinicalTrials.gov identifier: NCT04710420), a prospective, real-world, multicenter cohort derived from a Chinese peripheral arterial disease database (14). This study was conducted in accordance with the Declaration of Helsinki and approved by the local ethics committee. Written informed consent was obtained from each participant prior to the procedures. Between December 2020 and June 2023, consecutive patients were enrolled from 10 tertiary hospitals in mainland China, all of which were Class A (Grade 3) institutions with dedicated vascular surgery and/or endovascular centers.

The current analysis included diabetic patients with CLTI (Rutherford category 4–6) who had angiographically confirmed infrapopliteal (below-the-knee) atherosclerotic disease and underwent endovascular revascularization. Patients were excluded if they (1) were managed without endovascular therapy or underwent primary surgical revascularization, (2) did not receive infrapopliteal revascularization during the index procedure, or (3) lacked key baseline data or declined informed consent.

At baseline, demographic characteristics, comorbidities, and limb-specific findings were collected using standardized case report forms. WIfI staging was independently assessed by two investigators based on preprocedural clinical and imaging data, according to the SVS WIfI risk-of-amputation stages. Ischemia was graded primarily using ABI, with toe pressure or transcutaneous oxygen pressure (TcPO₂) used selectively when ABI was considered unreliable or noncompressible. Each limb was graded for wound (W), ischemia (I), and foot infection (fI), and the composite scores were mapped to WIfI stages 1–4. Detailed criteria are provided in Supplementary Data and Supplementary Figure S1.

### 2.2 Endovascular procedure and perioperative management

Endovascular treatment strategies—including target-vessel selection, management of inflow and outflow disease, and use of plain old balloon angioplasty (POBA), drug-coated devices, stents, or atherectomy—were determined by the treating operators based on clinical presentation and angiographic findings. Vasodilators were administered when necessary to relieve procedure-related vasospasm. Patients with tissue loss received coordinated postoperative wound care, including debridement, infection control, and advanced wound management when clinically indicated. Guideline-directed medical therapy was recommended for all patients, including antiplatelet therapy, statins, glucose control, and cardiovascular risk factor optimization.

### 2.3 Outcomes and follow-up

The primary endpoint was 12-month freedom from major adverse events (F-MAE). MAE was defined as a composite of all-cause mortality, major amputation, or clinically driven target-limb reintervention (CD-TLR); therefore, F-MAE represented the absence of any MAE component. Secondary endpoints included amputation-free survival (AFS), freedom from major amputation, overall survival, and freedom from CD-TLR, clinical improvement, and VascuQoL score change at 12 months.

Clinical improvement was assessed at the limb level as a binary outcome, defined as a ≥1-category improvement in Rutherford classification without repeat revascularization or major amputation during follow-up. Procedural success was defined as restoration of inline flow to the foot with no major complications within 1 week. Major complications within 1 week included cerebrovascular events, major bleeding, access-site complications requiring reintervention, peri-procedural death, and unplanned amputation; minor complications were those managed conservatively without reintervention. Quality of life was assessed at baseline and follow-up using the VascuQoL questionnaire. Standardized follow-up was conducted by telephone at 1, 6, and 12 months using structured assessments. Outpatient reassessment and further investigations were recommended when clinically indicated.

### 2.4 Statistical analysis

Categorical variables are presented as counts and percentages, and continuous variables as mean ± standard deviation. Time-to-event outcomes were analyzed using Kaplan–Meier methods, with comparisons performed using the log-rank test. Survival time was defined from the index endovascular procedure to the first occurrence of the endpoint of interest or the last completed follow-up. Cox proportional hazards regression was used to evaluate associations between WIfI stage and time-to-event outcomes. The interaction between diabetes status and WIfI stage for MAE was evaluated in the full PRIME-WIFI cohort. Multivariable models were fitted with adjustment for age, body mass index, hypertension, hyperlipidemia, atrial fibrillation, chronic kidney disease, preprocedural infrapopliteal runoff score, preprocedural pedal runoff score, calcification score, and infrapopliteal drug-coated balloon use. WIfI stage was modeled both as a categorical variable and as an ordinal per-stage variable. Pairwise stage comparisons were obtained by refitting models with alternative reference categories. Proportional hazards assumptions were assessed using Schoenfeld residuals. For limb-level major amputation with sparse events, Firth's penalized Cox regression was applied. Harrell's C-index was calculated to evaluate the discriminatory ability of WIfI stage for the time-to-event outcomes. Clinical improvement across WIfI stages was compared using the Pearson *χ*^2^ test. VascuQoL scores were compared among WIfI stages using one-way analysis of variance (ANOVA), and within-stage changes were assessed using paired t-tests. A repeated-measures ANOVA was used to compare the magnitude of change across WIfI stages. All tests were two-sided, with *P* < 0.05 considered statistically significant. Analyses were performed using R version 4.2.3 (The R Foundation for Statistical Computing, Vienna, Austria).

## 3 Results

### 3.1 Patient and limb characteristics

A total of 707 diabetic patients with 730 infrapopliteal CLTI limbs were included in the present study. In the full PRIME-WIFI cohort, interaction analysis showed a borderline interaction between diabetes status and WIfI stage for the primary outcome (*P* for interaction = 0.083). The mean age of the diabetic subgroup was 70.72 ± 10.20 years, and 65.63% of patients were male. Baseline demographic and clinical characteristics are summarized in Table 1. At 12 months, 92 of 707 patients (13.01%) were lost to follow-up, with 11 (13.25%), 19 (12.50%), 38 (15.20%), and 24 (10.81%) patients in WIfI stages 1–4, respectively. Overall, the comorbidity burden was high, with hypertension, coronary artery disease, and chronic renal insufficiency being highly prevalent. Baseline quality of life was markedly impaired. A history of prior peripheral artery disease treatment was documented in nearly one-third of patients, and one-fifth had undergone previous target-limb revascularization.

**Table 1:** Patient characteristics.

Variables	Patients (*n* = 707)
Age, years	70.719 ± 10.201
BMI, kg/m^2^	22.791 ± 3.315
VascuQoL score	2.344 ± 1.130
Laterality	
Bilateral	23 (3.25%)
Right	339 (47.95%)
Left	345 (48.80%)
Male	464 (65.63%)
Smoking history	175 (24.75%)
Coronary artery disease	209 (29.56%)
History of cerebral infarction	162 (22.91%)
COPD	14 (1.98%)
Hypertension	535 (75.67%)
Atrial fibrillation	19 (2.69%)
Diabetes mellitus	707 (100%)
Chronic renal insufficiency	117 (16.55%)
History of PAD treatment	227 (32.11%)
History of target limb revascularization	143 (20.23%)
Open surgery	5 (0.71%)
Endovascular	140 (19.80%)

At the limb level, baseline characteristics are detailed in Table 2. Most limbs presented with severe disease, with a predominance of Rutherford category 5. Limbs were distributed across all four WIfI stages, with a clear predominance of higher-risk stages (stages 3 and 4). An ABI >1.40 was recorded in 32 of the 730 limbs. Preprocedural infrapopliteal and pedal runoff assessments reflect severely compromised distal perfusion. Preprocedural runoff scores and disease extent by WIfI stage are presented in Supplementary Table S2.

**Table 2:** Limb characteristics.

Variables	Limbs (*n* = 730)
Rutherford category	
4	79 (10.82%)
5	578 (79.18%)
6	73 (10.00%)
WIfI wound grade	
0	79 (10.82%)
1	406 (55.62%)
2	155 (21.23%)
3	90 (12.33%)
WIfI ischemia grade	
0	166 (22.74%)
1	85 (11.64%)
2	180 (24.66%)
3	299 (41.00%)
WIfI foot infection grade	
0	174 (23.84%)
1	357 (48.90%)
2	191 (26.16%)
3	8 (1.10%)
WIfI stage	
Stage 1	91 (12.47%)
Stage 2	160 (21.92%)
Stage 3	257 (35.21%)
Stage 4	222 (30.41%)
Number of diseased infrapopliteal arteries	
1	59 (8.08%)
2	188 (25.75%)
3	483 (66.16%)
Preprocedural infrapopliteal runoff score	10.117 ± 3.876
Preprocedural pedal runoff score	2.127 ± 0.699

### 3.2 Lesion and procedural characteristics

Overall, 1,383 infrapopliteal target lesions were treated (Table 3). Lesions were diffuse and complex, with a high burden of calcification and a substantial proportion of chronic total occlusions. With respect to endovascular strategy, POBA and drug-coated balloon (DCB) angioplasty were predominantly used, while stenting and atherectomy were performed selectively. Concomitant inflow intervention was performed in 60.41% of limbs and outflow intervention in 21.37%.

**Table 3:** Infrapopliteal lesion and procedural characteristics.

Variables	*N*
Targeted infrapopliteal lesions	*N* = 1,383
Lesion location	
Popliteal artery P3	171 (12.36%)
Anterior tibial artery	453 (32.75%)
Posterior tibial artery	340 (24.58%)
Peroneal artery	315 (22.78%)
Tibioperoneal trunk	104 (7.52%)
Calcification	
1	305 (22.05%)
2	627 (45.34%)
3	266 (19.23%)
4	185 (13.38%)
Lesion type	
*de novo* lesion	1,322 (95.59%)
ISR or Restenotic lesion	61 (4.41%)
Chronic total occlusion lesion	905 (65.44%)
Infrapopliteal intervention	*N* = 730
POBA alone	346 (47.40%)
DCB angioplasty	349 (47.81%)
Stenting	53 (7.26%)
Atherectomy	14 (1.92%)
Concomitant inflow intervention	441 (60.41%)
Distal abdominal aorta	2 (0.27%)
Iliac artery	18 (2.47%)
Femoral artery	383 (52.47%)
Popliteal artery P1–2	323 (44.25%)
Concomitant outflow intervention	156 (21.37%)
Postprocedural infrapopliteal runoff score	4.87 ± 2.34
Postprocedural pedal runoff score	1.83 ± 0.86
Procedural success	716 (98.08%)
Perioperative complications (≤1 week)	28 (3.84%)
Minor complications	17 (2.33%)
Major complications	11 (1.51%)

The procedural success rate was 98.08%. Both infrapopliteal and pedal runoff scores improved significantly after intervention (all *P* < 0.001). Early complications within 1 week occurred in 3.84% of cases, with major complications in 1.51%, including 4 cerebrovascular accidents, 2 major bleeding events, 2 pseudoaneurysms requiring reintervention, 2 deaths, and 1 unplanned amputation.

### 3.3 F-MAE

A total of 156 MAE events were observed during follow-up. Kaplan–Meier analysis demonstrated lower F-MAE with increasing WIfI stage, with overlapping curves between stages 2 and 3 (Figure 1). One-year F-MAE rates were 89.5% (82.9–96.7%) for stage 1, 77.4% (70.9–84.5%) for stage 2, 78.0% (72.8–83.6%) for stage 3, and 70.1% (64.2–76.5%) for stage 4 (log-rank *P* = 0.004). In multivariable Cox regression analysis adjusted for age, body mass index, hypertension, hyperlipidemia, atrial fibrillation, chronic kidney disease, infrapopliteal runoff score, preprocedural pedal runoff score, calcification score, and infrapopliteal drug-coated balloon use, WIfI stage remained independently associated with MAE, with each 1-stage increase corresponding to a higher hazard of MAE events (HR: 1.31, 95% CI: 1.09–1.56; *P* = 0.0031). In multivariable stage-wise comparisons, the hazard of MAE events was significantly higher for stage 4 vs. stage 1 (HR: 2.97, 95% CI: 1.41–6.24; *P* = 0.004) and stage 4 vs. stage 3 (HR: 1.62, 95% CI: 1.11–2.37; *P* = 0.013), whereas stage 2 vs. stage 1 showed a borderline association (HR: 2.12, 95% CI: 0.97–4.63; *P* = 0.059) and the remaining pairwise comparisons were not statistically significant (Supplementary Table S1A). The Harrell's C-index of WIfI stage for MAE was 0.581, indicating limited discrimination.

**Figure 1:**
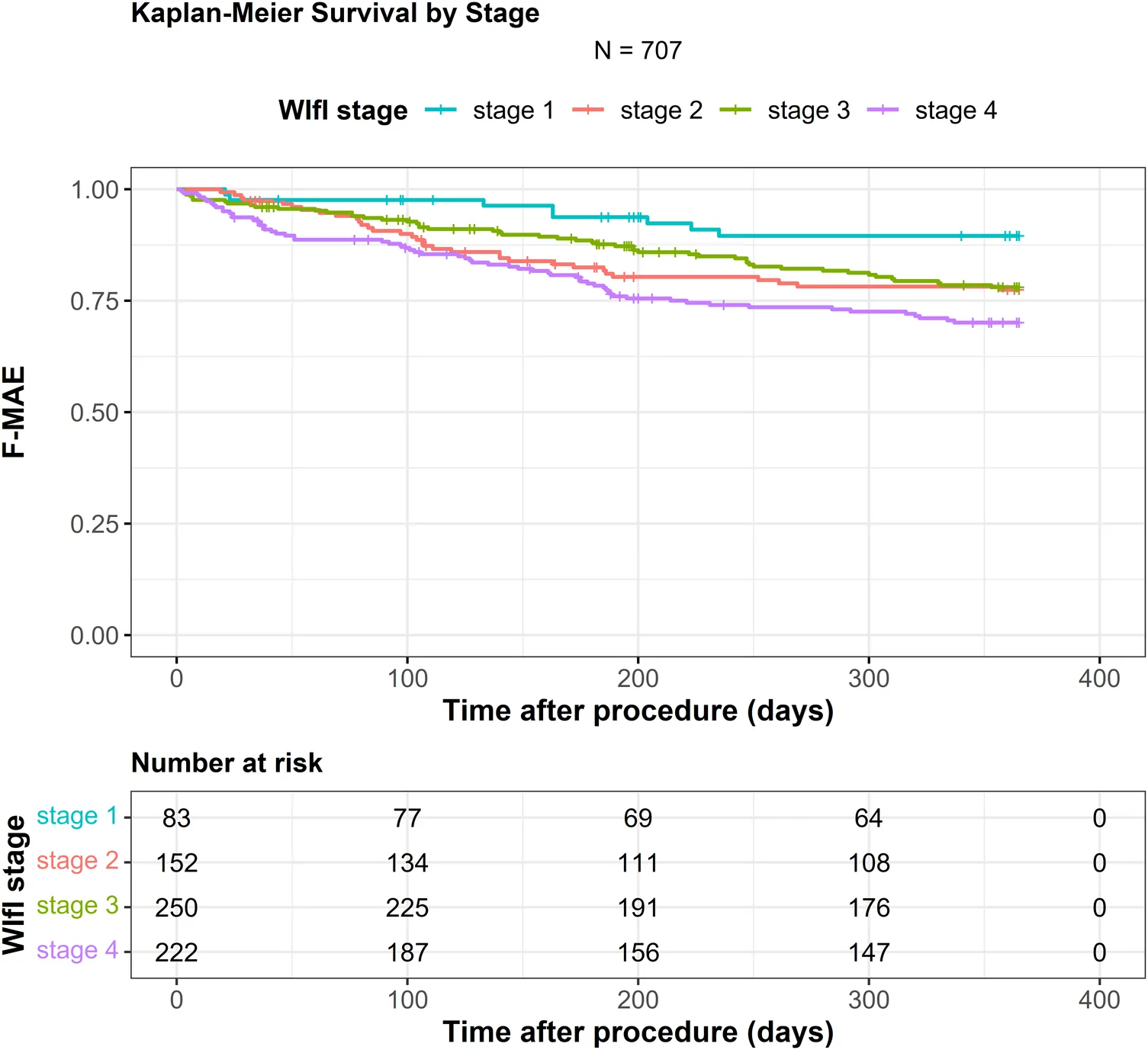
Kaplan–Meier estimates of freedom from major adverse events (F-MAE) by WIfI stage. The association was statistically significant (log-rank *P* = 0.004).

### 3.4 AFS

During follow-up, 121 AFS events were observed. Kaplan–Meier analysis showed a decline in AFS rate with increasing WIfI stage, with considerable overlap between stages 2 and 3 (Figure 2). The estimated 1-year AFS rates were 94.7% (89.8–99.9%) for stage 1, 83.3% (77.5–89.7%) for stage 2, 83.3% (78.6–88.3%) for stage 3, and 74.2% (68.5–80.3%) for stage 4 (log-rank *P* = 0.0003). After multivariable adjustment using the same covariate adjustment, WIfI stage remained independently associated with AFS, with each 1-stage increase corresponding to a higher hazard of major amputation or death (HR: 1.53, 95% CI: 1.24–1.89; *P* = 8.53 × 10^−5^). In multivariable stage-wise comparisons, significant differences were observed for stage 2 vs. stage 1 (HR: 2.99, 95% CI: 1.03–8.69; *P* = 0.045), stage 4 vs. stage 1 (HR: 5.42, 95% CI: 1.95–15.07; *P* = 0.001), stage 4 vs. stage 2 (HR: 1.82, 95% CI: 1.11–2.96; *P* = 0.017), and stage 4 vs. stage 3 (HR: 1.94, 95% CI: 1.27–2.97; *P* = 0.002). The comparison between stage 3 and stage 1 showed a borderline association (HR: 2.79, 95% CI: 0.99–7.90; *P* = 0.053), whereas stage 3 did not differ significantly from stage 2 (Supplementary Table S1B). The Harrell's C-index of WIfI stage for AFS was 0.606.

**Figure 2:**
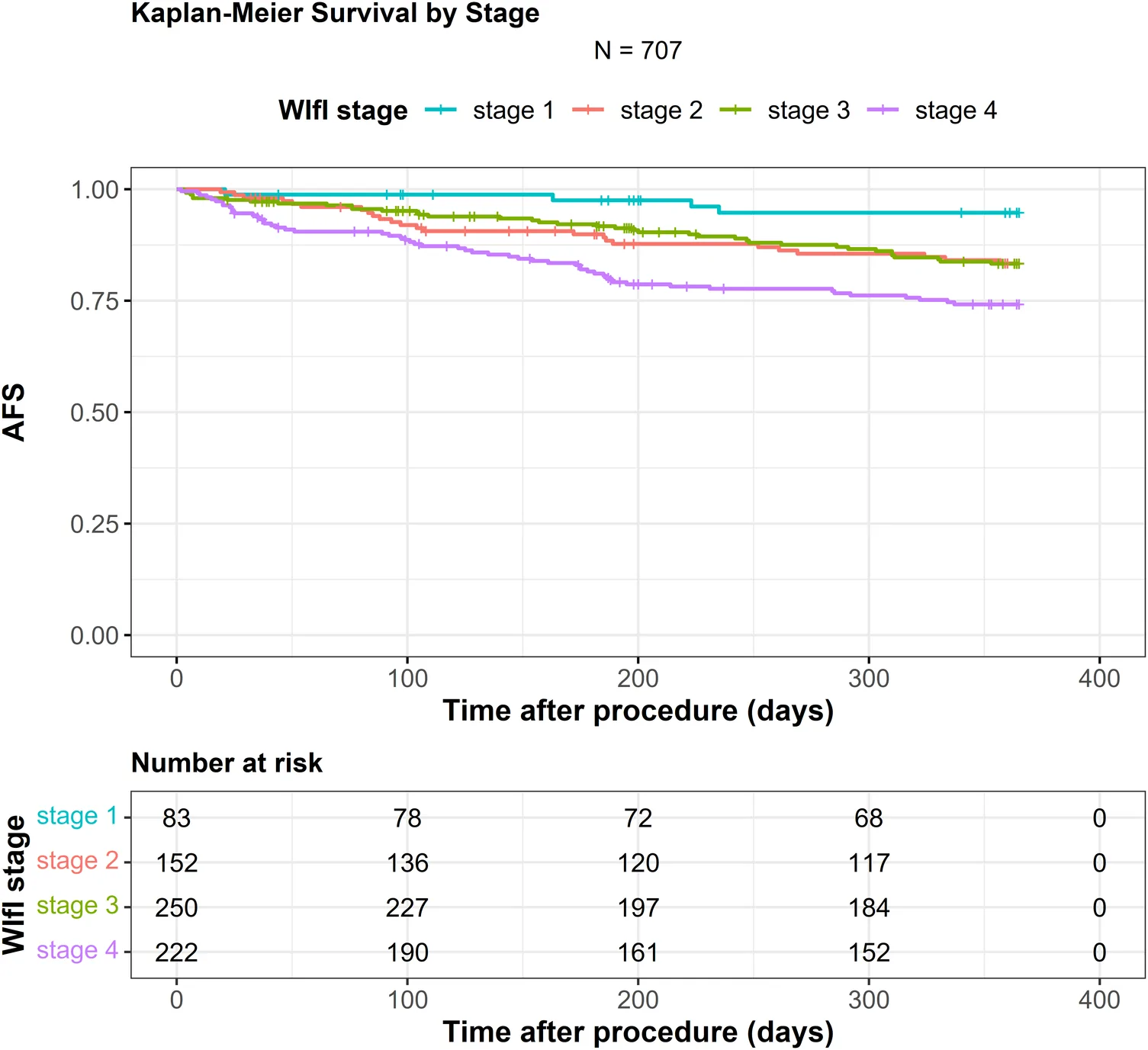
Kaplan–Meier estimates of amputation-free survival (AFS) by WIfI stage. The association was statistically significant (log-rank *P* = 0.0003).

### 3.5 Survival

During follow-up, a total of 93 deaths were recorded. Kaplan–Meier analysis demonstrated lower overall survival with increasing WIfI stage, with a similar overlap between stages 2 and 3 observed (Figure 3). The estimated 1-year overall survival rates were 94.7% (89.8–99.9%) for stage 1, 87.8% (82.5–93.4%) for stage 2, 86.1% (81.7–90.8%) for stage 3, and 80.3% (75.0–85.9%) for stage 4 (log-rank *P* = 0.009). In multivariable Cox regression analysis using the same covariate adjustment, WIfI stage was independently associated with all-cause mortality, with each 1-stage increase corresponding to a higher hazard of death (HR: 1.52, 95% CI: 1.20–1.94; *P* = 0.0007). In multivariable pairwise comparisons, significant differences were observed for stage 4 vs. stage 1 (HR: 4.17, 95% CI: 1.48–11.79; *P* = 0.007), stage 4 vs. stage 2 (HR: 2.01, 95% CI: 1.13–3.58; *P* = 0.018), and stage 4 vs. stage 3 (HR: 1.87, 95% CI: 1.16–3.02; *P* = 0.010), whereas the remaining pairwise comparisons were not statistically significant (Supplementary Table S1C). Harrell's C-index of WIfI stage for survival was 0.598.

**Figure 3:**
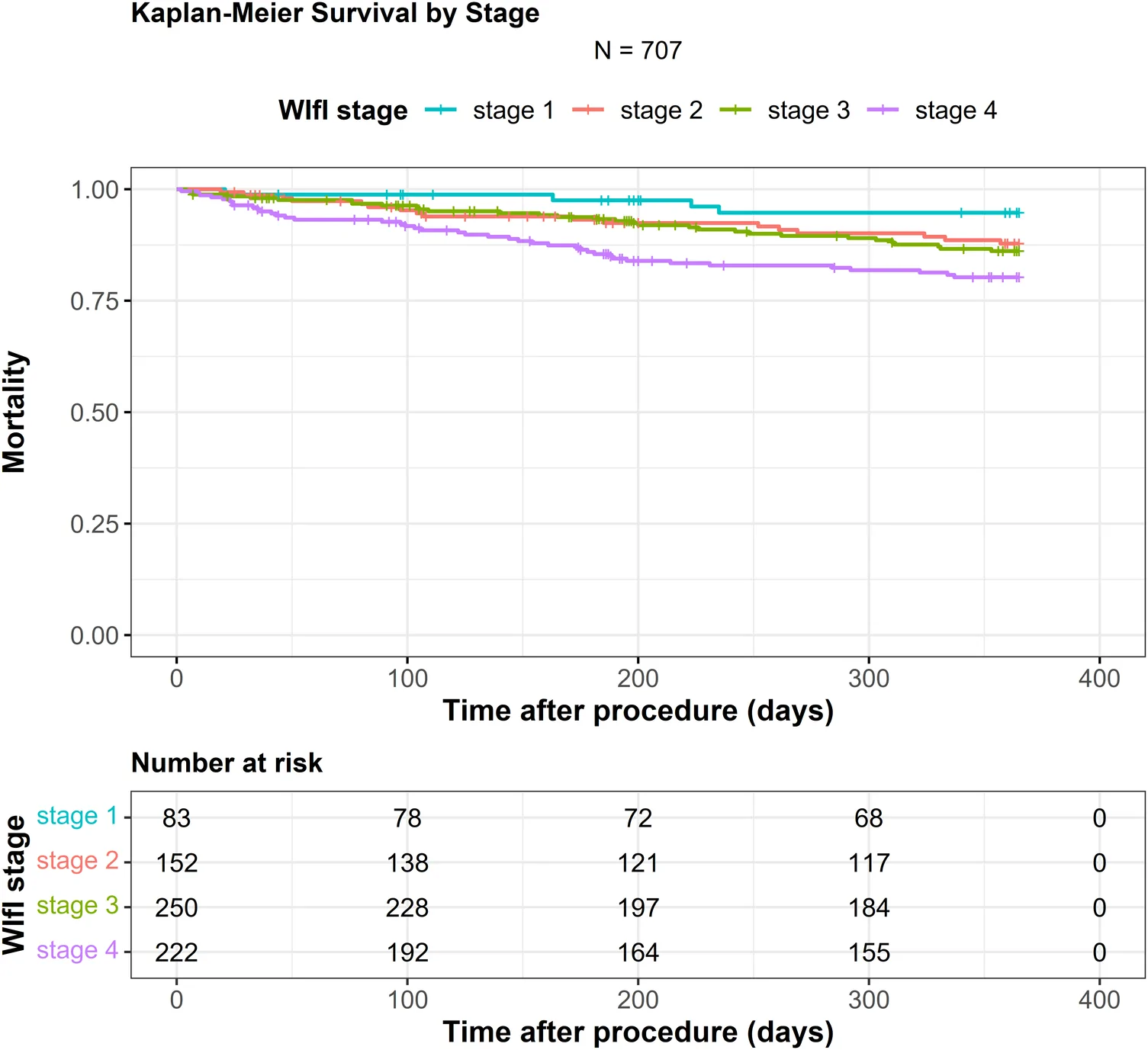
Kaplan–Meier estimates of overall survival by WIfI stage. The association was statistically significant (log-rank *P* = 0.009).

### 3.6 Major amputations

A total of 29 major amputations were observed during follow-up. Kaplan–Meier analysis showed decreasing freedom from major amputation with increasing WIfI stage, with 100.0% for stage 1, 95.3% (91.9–98.8%) for stage 2, 96.8% (94.5–99.2%) for stage 3, and 92.3% (88.6–96.2%) for stage 4 (log-rank *P* = 0.02) (Figure 4). Given the absence of events in stage 1, Firth's penalized Cox regression was used for multivariable analyses. Each 1-stage increase in WIfI stage was associated with a higher hazard of major amputation (HR: 1.62, 95% CI: 1.07–2.57; *P* = 0.020). Exploratory stage-wise comparisons yielded wide confidence intervals because of sparse events, but stage 4 remained associated with higher risk than stage 3 (HR: 2.48, 95% CI: 1.05–6.39; *P* = 0.039) (Supplementary Table S1D). The Harrell's C-index of WIfI stage for major amputation was 0.665.

**Figure 4:**
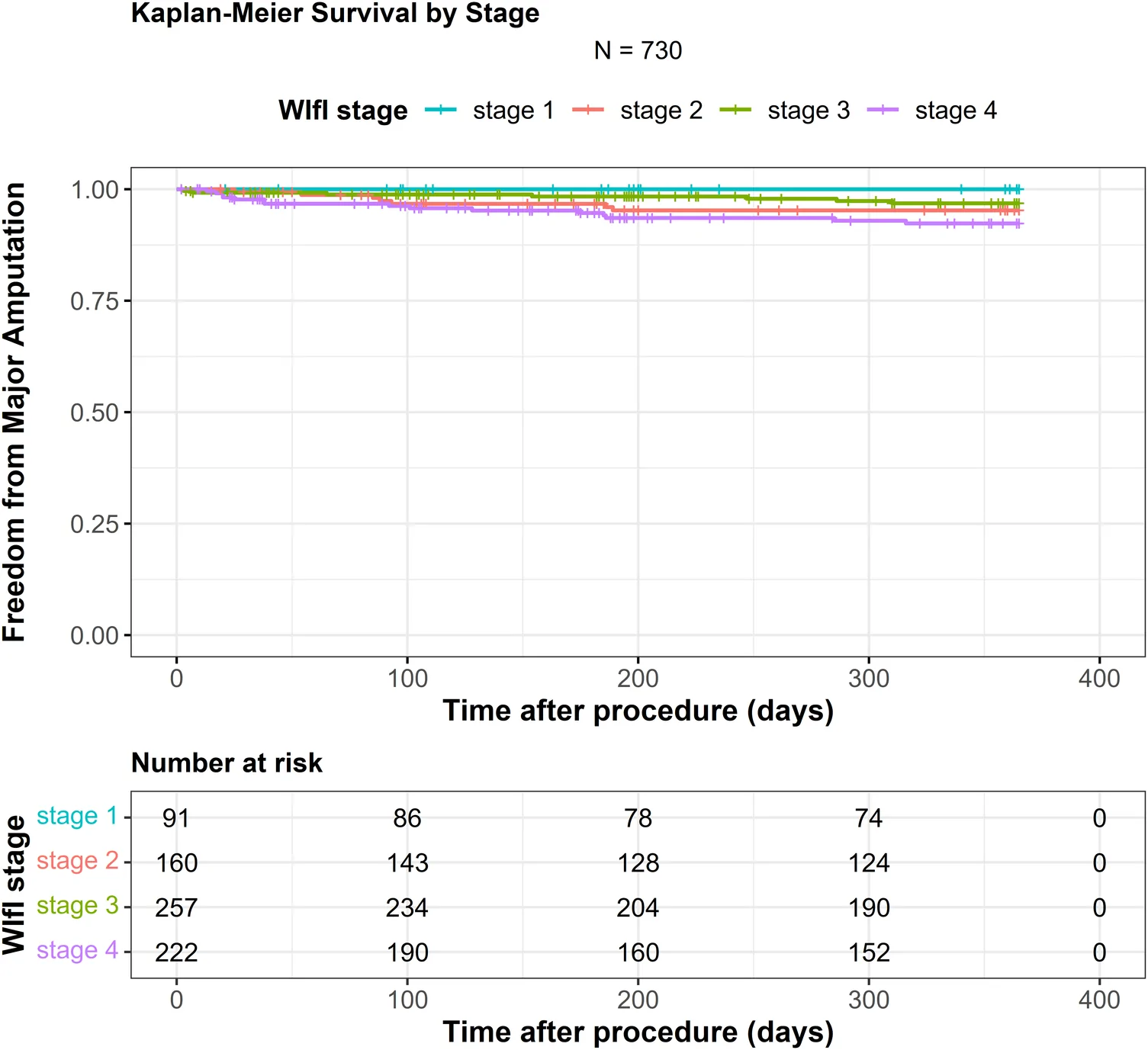
Kaplan–Meier estimates of freedom from major amputation by WIfI stage. The association was statistically significant (log-rank *P* = 0.02).

### 3.7 CD-TLR

A total of 45 CD-TLR events were observed during follow-up. Kaplan–Meier curves were largely overlapping across WIfI stages, with 12-month freedom from CD-TLR of 95.2% (90.7–99.9%) for stage 1, 91.2% (86.7–95.9%) for stage 2, 92.4% (89.0–96.0%) for stage 3, and 94.1% (90.8–97.6%) for stage 4 (log-rank *P* = 0.60) (Figure 5). In multivariable Cox regression with the same covariate adjustment, WIfI stage was not independently associated with CD-TLR (per-stage HR: 0.92, 95% CI: 0.68–1.23; *P* = 0.564), and no pairwise stage-wise comparison reached statistical significance (all *P* > 0.05) (Supplementary Table S1E). The Harrell's C-index of WIfI stage for CD-TLR was 0.561.

**Figure 5:**
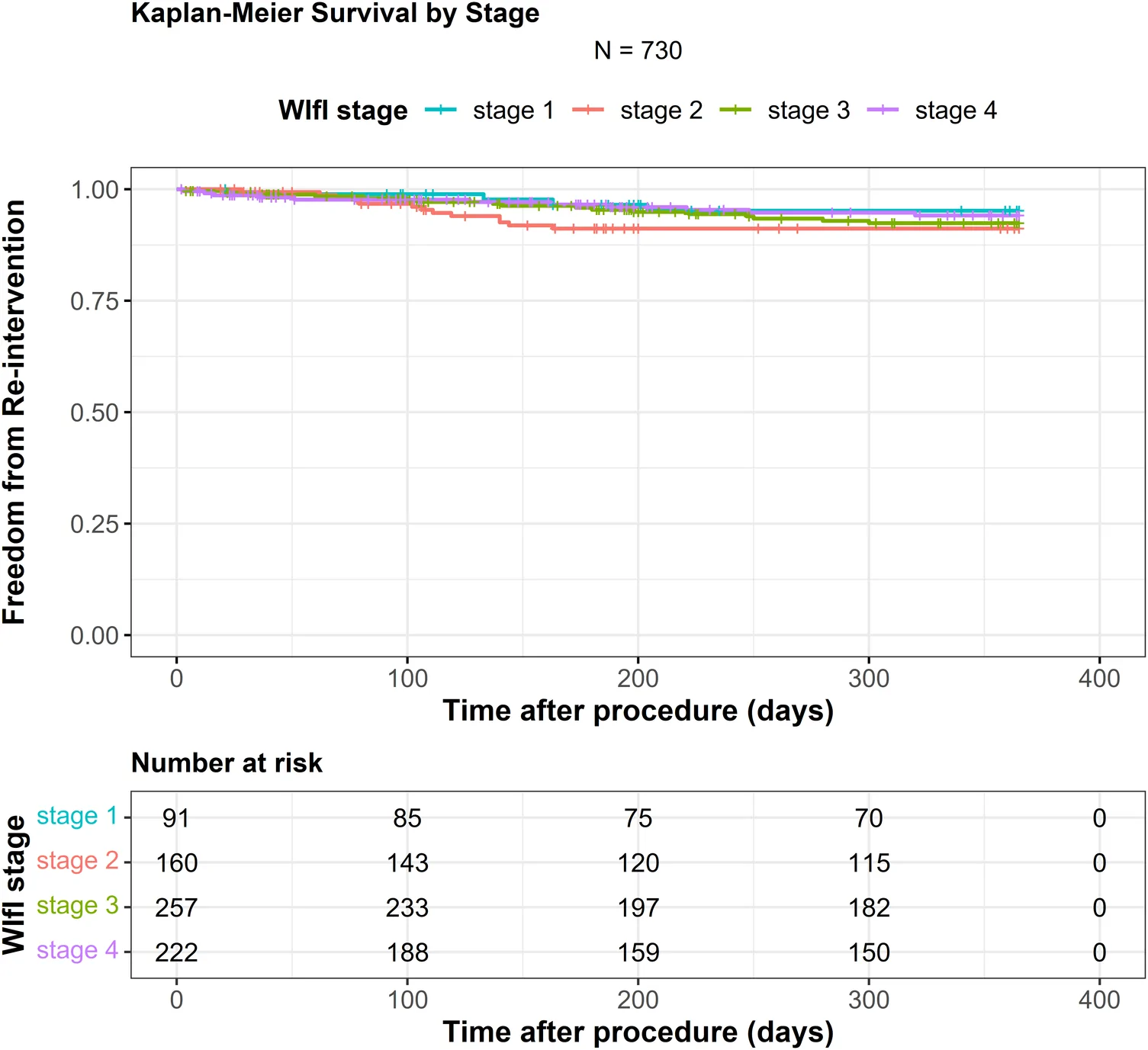
Kaplan–Meier estimates of freedom from clinically driven target-limb reintervention (CD-TLR) by WIfI stage. The association was not statistically significant (log-rank *P* = 0.60).

### 3.8 Clinical improvement and quality of life

Clinical improvement was assessed at the limb level across WIfI stages (Figure 6). Overall, the likelihood of clinical improvement tended to decrease with increasing WIfI stage; however, an overlap and partial reversal between stages 2 and 3 was observed. The overall difference in clinical improvement across WIfI stages did not reach statistical significance, although a borderline trend was noted (Pearson *χ*^2^ test, *P* = 0.053).

**Figure 6:**
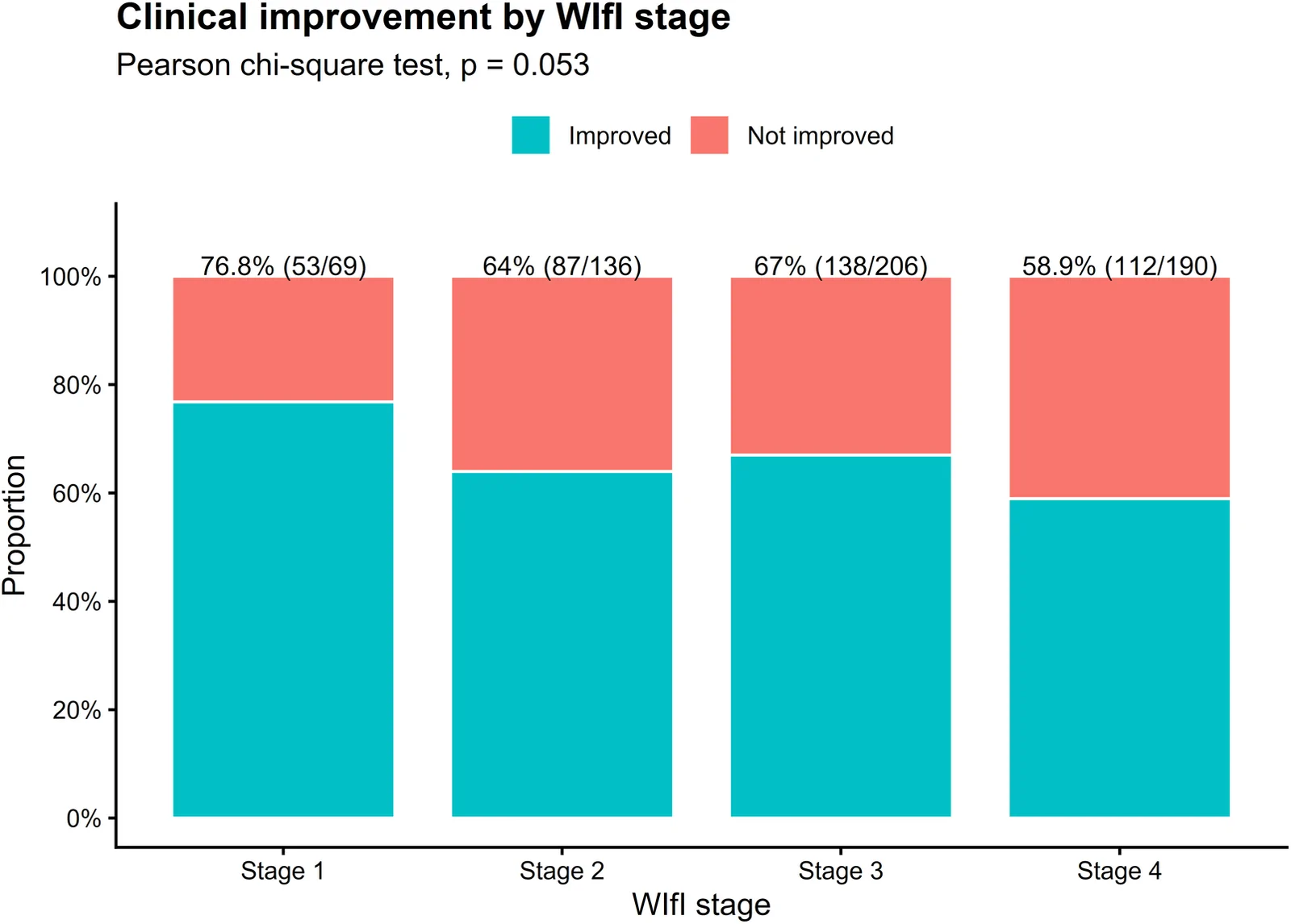
Proportion of limbs achieving clinical improvement by WIfI stage. The association did not reach statistical significance, although a borderline trend was observed (Pearson *χ*^2^ *P* = 0.053).

Quality of life was assessed using the VascuQoL questionnaire (Figure 7). Baseline VascuQoL scores differed significantly across WIfI stages (*P* < 0.001). At 12 months, the mean VascuQoL scores were 5.01 ± 1.57, 4.33 ± 2.12, 4.02 ± 2.07, and 3.99 ± 2.37 in WIfI stages 1–4, respectively, and remained significantly different across stages (*P* < 0.001). However, the magnitude of improvement did not differ significantly among stages, as assessed by repeated-measures ANOVA (*P* for interaction = 0.247).

**Figure 7:**
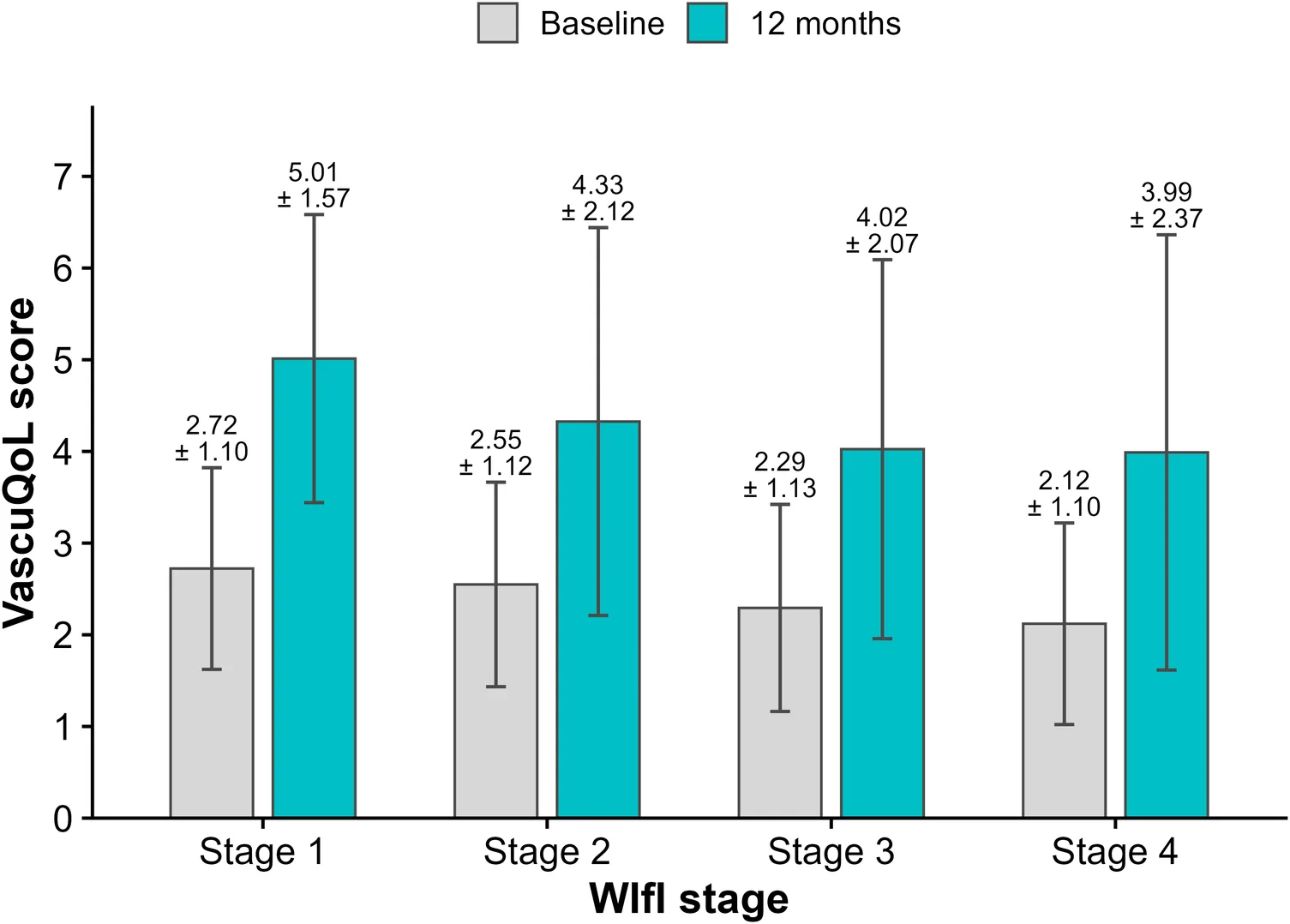
VascuQoL scores at baseline and 12 months according to WIfI stage. Bars represent mean values, and error bars represent standard deviations. Between-stage comparisons were performed using one-way ANOVA, and changes over time among WIfI stages were evaluated using repeated-measures ANOVA.

## 4 Discussion

In this diabetic sub-cohort of the PRIME-WIFI registry, several important findings emerged. First, WIfI stage retained an overall prognostic gradient for major clinical outcomes following infrapopliteal endovascular revascularization in patients with CLTI and diabetes mellitus, including F-MAE, AFS, overall survival, and major amputation, but not freedom from CD-TLR. Second, although higher WIfI stages were consistently associated with worse outcomes, the discriminative separation between intermediate stages—particularly stages 2 and 3—was attenuated, with overlapping survival curves, limited pairwise differences, and modest Harrell's C-index values across endpoints. Third, clinical improvement showed only a borderline association with WIfI stage, while VascuQoL scores improved across all stages without a significant difference in the magnitude of improvement among stages. Collectively, these findings suggest that WIfI stage remains a useful global risk-stratification tool in diabetic patients with infrapopliteal CLTI undergoing endovascular revascularization, although its discriminatory granularity is reduced among intermediate-risk groups.

Numerous studies have established the WIfI staging as a prognostic framework in CLTI. A systematic review and meta-analysis demonstrated progressively higher major amputation risk and lower AFS with increasing WIfI stage, while subsequent studies in revascularized populations, including the BEST-CLI trial, broadly confirmed its association with adverse limb outcomes and mortality (15–19). However, much of the available evidence has been limited by retrospective designs, modest sample sizes, or heterogeneity in patient populations and treatment strategies. To address these limitations, the prospective, multicenter PRIME-WIFI registry evaluated WIfI staging in a contemporary real-world cohort of patients with infrapopliteal CLTI undergoing endovascular therapy. In the overall PRIME-WIFI cohort, preoperative WIfI stage was independently associated with F-MAE, AFS, major amputation, and all-cause mortality, although the observed risk separation was driven predominantly by stage 4 (14). Taken together, these findings support the prognostic utility of WIfI staging in revascularized CLTI populations while underscoring the need to evaluate its performance in high-risk subgroups, particularly patients with diabetes mellitus.

As a highly prevalent comorbidity in patients with CLTI, diabetes mellitus serves as a critical disease modifier that adversely affects prognosis across most major clinical endpoints. This association is likely mediated by more severe tissue loss and infection at presentation, a higher burden of systemic comorbidities, and diabetes-related microvascular dysfunction within a proinflammatory milieu (7, 20, 21). Consistent with this, multiple studies have reported higher risks of major amputation, all-cause mortality, and impaired wound healing among diabetic patients undergoing revascularization (6, 8, 22). These biological and anatomical complexities may also compromise the performance of the WIfI classification in this high-risk population.

Among the three WIfI domains, the ischemia (I) component appears particularly vulnerable to misclassification in diabetic limbs. Medial arterial calcification and vessel incompressibility may result in falsely elevated ABI values, leading to underestimation of ischemic severity (8, 23, 24). This limitation underscores the importance of corroborating ischemia severity when ABI is noncompressible or inconsistent with the clinical presentation. In such settings, toe pressure/TBI or TcPO₂ may provide complementary objective perfusion information and reduce uncertainty in WIfI ischemia grading (1, 20). In addition, macrovascular revascularization does not necessarily translate into adequate tissue perfusion in the presence of diabetes-related small artery disease and microcirculatory dysfunction, which are not fully captured by the current WIfI classification (20, 23). Finally, chronic hyperglycemia also impairs wound healing and immune response, potentially amplifying the risk associated with any given WIfI stage (25).

Despite these challenges, available evidence generally supports the prognostic relevance of WIfI staging in diabetic populations. Prior studies have shown that increasing WIfI stage is associated with higher risks of amputation and mortality in patients with diabetes-related limb threat (12, 13, 26). However, the existing data remain limited, as most reports are derived from retrospective analyses or cohorts with modest sample sizes and heterogeneous clinical contexts. Consequently, the strength and consistency of evidence supporting WIfI performance specifically in revascularized diabetic CLTI patients remain less well established, underscoring the need for further evaluation in contemporary, real-world cohorts. The present analysis extends the parent PRIME-WIFI findings by evaluating WIfI within a large, prospectively enrolled diabetic subgroup, formally assessing the WIfI-by-diabetes interaction for MAE, and quantifying within-subgroup discrimination using pairwise comparisons and Harrell's C-indices. Although the interaction for MAE was borderline and should be considered hypothesis-generating rather than conclusive, WIfI showed a consistent overall prognostic gradient but only modest discrimination within the diabetic subgroup.

An important distinction between the present diabetic subgroup and the overall PRIME-WIFI cohort lies in the pattern of risk separation across WIfI stages. Whereas prognostic differences in the overall PRIME-WIFI cohort were driven predominantly by stage 4, the clearest separation in the diabetic subgroup was observed between the extremes of the WIfI spectrum, with stage 1 showing the most favorable outcomes and stage 4 the poorest, while stages 2 and 3 substantially overlapped. This pattern suggests that, in diabetic CLTI, WIfI may be more useful for broad risk stratification than for providing granular discrimination across all four stages. However, because the interaction between diabetes status and WIfI stage did not reach conventional statistical significance, this pattern should be considered hypothesis-generating and cannot be attributed specifically to diabetes. This limited separation among intermediate or lower WIfI stages has been reported in previous studies and reviews (17, 27, 28). Several factors may explain this finding. First, ischemia grading in this registry relied predominantly on ABI, which may underestimate ischemic severity and contribute to stage overlap. In diabetic patients, microvascular dysfunction, infection burden, and impaired wound healing may further contribute to this overlap. Second, WIfI was originally developed to stratify limb threat at presentation, and preprocedural staging may be less granular for predicting post-revascularization outcomes (29). Third, composite WIfI stages may include heterogeneous combinations of wound, ischemia, and infection grades, which may carry different biological and prognostic implications despite being assigned to adjacent clinical stages.

Additionally, WIfI stage was not significantly associated with CD-TLR, which may reflect the influence of factors beyond baseline limb threat, including anatomical disease burden, vessel durability, procedural strategy, and clinical decision-making. Mean runoff scores and three-vessel disease extent did not increase monotonically across WIfI stages, suggesting that WIfI stage does not fully capture anatomical features potentially relevant to reintervention. Similar findings have been reported in other CLTI studies (30, 31). Likewise, although Rutherford-based improvement captures only one dimension of recovery, its borderline stage-wise association with WIfI still suggests that recovery after revascularization is influenced by factors beyond baseline limb-threat severity.

Taken together, these limitations suggest that WIfI should not be interpreted as a standalone framework for comprehensive post-revascularization risk assessment. Rather, its value lies in providing a structured assessment of baseline limb threat, which represents only one dimension of the subsequent clinical course (32). This distinction may be particularly relevant in diabetic CLTI, in which the relationship between baseline limb-threat severity and recovery after revascularization may be complicated by diabetes-related alterations in tissue perfusion and healing. Accordingly, WIfI may be more appropriately used as a common framework for characterizing limb severity, while interpretation of subsequent risk and recovery should remain grounded in the evolving clinical condition of the patient. In this context, WIfI can be incorporated into multidisciplinary limb-salvage pathways as one component of longitudinal assessment rather than used in isolation (1, 33).

## 5 Limitations

Several limitations should be acknowledged. First, as a subgroup analysis derived from a prospective multicenter registry rather than an independently powered cohort, this study may be subject to residual confounding and reduced statistical power; the 13.01% loss to follow-up may additionally have introduced attrition bias. Also, the glycemic control could not be adequately assessed because HbA1c was not systematically collected in the registry. Second, ischemia grading relied predominantly on ABI, which may lead to underestimation of ischemic severity in diabetic patients, while toe pressure and TcPO₂ were collected selectively rather than systematically. Third, the exclusive inclusion of tertiary vascular centers in a single country may limit the generalizability of the findings to other healthcare settings. Finally, clinical improvement was assessed using Rutherford category changes, which may not fully reflect wound healing, functional recovery, or broader patient-centered recovery in diabetic patients.

## 6 Conclusion

In this large diabetic subgroup of the PRIME-WIFI registry with infrapopliteal CLTI undergoing endovascular revascularization, WIfI stage remained associated with MAE, AFS, mortality, and major amputation, but not CD-TLR. However, discrimination was clearest between stages 1 and 4, with substantial overlap between stages 2 and 3. These findings refine the overall PRIME-WIFI results by defining WIfI performance specifically in diabetic patients and support its use for broad, rather than granular, risk stratification.

## Data Availability

The raw data supporting the conclusions of this article will be made available by the authors, without undue reservation.
