# Supplementary material for: Prognostic performance of the WIfI classification in diabetic patients with infrapopliteal CLTI after endovascular revascularization: a subgroup analysis of the PRIME-WIFI registry
Source: Front Cardiovasc Med. 2026 Oct 29;13:1984270. doi: 10.3389/fcvm.2026.1984270 (PMC13754645; doi:10.3389/fcvm.2026.1984270)
Supplement: Supplementary file 1 [file Table1.docx]

***Supplementary Material***

# 1 Supplementary Data

## 1.1 WIfI Classification

Photographs of wounds from multiple angles were taken for all patients with ulcers before treatment and uploaded to the database. The Wound, Ischemia, and foot Infection (WIfI) classification assessment was performed independently by two investigators; in cases of discrepancy, a third investigator was consulted for adjudication. The resulting WIfI combination was then assigned to a clinical stage from 1 to 4 according to the Society for Vascular Surgery WIfI clinical-stage matrix for estimated 1-year major amputation risk.(1)

### 1.1.1 Wound (W, Grade 0–3)

1)	Grade 0: No ulcer and no gangrene.

2)	Grade 1: Small, shallow ulcer(s) on the distal leg or foot; no exposed bone, unless exposure is limited to the distal phalanx; no gangrene.

3)	Grade 2: Deeper ulcer with exposed bone, joint, or tendon; generally not involving the heel; or a shallow heel ulcer without calcaneal involvement; or gangrenous changes limited to digits.

4)	Grade 3: Extensive, deep ulcer involving the forefoot and/or midfoot; deep, full-thickness heel ulcer ± calcaneal involvement; or extensive gangrene involving forefoot and/or midfoot; full-thickness heel necrosis ± calcaneal involvement.

### 1.1.2 Ischemia grade (I, Grade 0–3)

In the PRIME-WIFI registry, ischemia grading was primarily based on ankle-brachial index (ABI). Toe pressure and transcutaneous oxygen pressure (TcPO2) were used to refine ischemia assessment when ABI was considered unreliable or noncompressible.

1)	Grade 0: ABI ≥0.80 or toe pressure/TcPO₂ ≥60 mmHg;

2)	Grade 1: ABI 0.60–0.79 or toe pressure/TcPO₂ 40–59 mmHg;

3)	Grade 2: ABI 0.40–0.59 or toe pressure/TcPO₂ 30–39 mmHg;

4)	Grade 3: ABI ≤0.39 or toe pressure/TcPO₂ <30 mmHg.

### 1.1.3 foot Infection (fI, Grade 0–3)

1)	Grade 0: No signs or symptoms of infection. Infection is defined by the presence of at least 2 of the following items: local swelling or induration; erythema ＞0.5 to ≤2 cm around the ulcer; local tenderness or pain; local warmth; purulent discharge (thick, opaque to white, or sanguineous secretion).

2)	Grade 1: Mild, local infection involving only the skin and subcutaneous tissue (no systemic signs; limited and superficial). Exclude other causes of an inflammatory response of the skin (eg, trauma, gout, fracture, thrombosis, venous stasis).

3)	Grade 2: Moderate infection — deeper or more extensive local infection (e.g., deeper tissues such as muscle, tendon, or joint) but without systemic inflammatory response; or infection with local spread.

4)	Grade 3: Severe infection — systemic inflammatory response syndrome or sepsis; systemic signs of infection and/or life- or limb-threatening infection.

### 1.1.4 WIfI clinical stage assignment

The matrix below shows how combinations of W, I, and fI grades were assigned to WIfI clinical stages for estimated 1-year major amputation risk. Stage 1 indicates very low risk, stage 2 low risk, stage 3 moderate risk, and stage 4 high risk. Clinical stage 5 indicates an unsalvageable foot and was not included in the present matrix.

| **Ischemia grade 0** **W/fI** \| **0** \| **1** \| **2** \| **3**; **W 0** \| **1** \| **1** \| **2** \| **3**; **W 1** \| **1** \| **1** \| **2** \| **3**; **W 2** \| **2** \| **2** \| **3** \| **4**; **W 3** \| **3** \| **3** \| **4** \| **4** | | **Ischemia grade 1** **W/fI** \| **0** \| **1** \| **2** \| **3**; **W 0** \| **1** \| **2** \| **3** \| **4**; **W 1** \| **1** \| **2** \| **3** \| **4**; **W 2** \| **3** \| **3** \| **4** \| **4**; **W 3** \| **4** \| **4** \| **4** \| **4** | |
| --- | --- | --- | --- |
| **Ischemia grade 2** **W/fI** \| **0** \| **1** \| **2** \| **3**; **W 0** \| **2** \| **2** \| **3** \| **4**; **W 1** \| **2** \| **3** \| **4** \| **4**; **W 2** \| **3** \| **4** \| **4** \| **4**; **W 3** \| **4** \| **4** \| **4** \| **4** | | **Ischemia grade 3** **W/fI** \| **0** \| **1** \| **2** \| **3**; **W 0** \| **2** \| **3** \| **3** \| **4**; **W 1** \| **3** \| **3** \| **4** \| **4**; **W 2** \| **4** \| **4** \| **4** \| **4**; **W 3** \| **4** \| **4** \| **4** \| **4** | |
| **Stage 1: very low** | **Stage 2: low** | **Stage 3: moderate** | **Stage 4: high** |

**Supplementary Figure S1.** Society for Vascular Surgery WIfI clinical-stage matrix for estimated 1-year major amputation risk.

Abbreviations: fI, foot infection; W, wound.

# 2 Supplementary Tables

**Supplementary Table S1.** Adjusted pairwise comparisons between baseline WIfI stages for clinical outcomes

**S1A. MAE**

| **Comparison** | **Adjusted HR (95% CI)** | ***P* value** |
| --- | --- | --- |
| Stage 2 vs Stage 1 | 2.12 (0.97–4.63) | 0.059 |
| Stage 3 vs Stage 1 | 1.83 (0.86–3.91) | 0.116 |
| Stage 4 vs Stage 1 | 2.97 (1.41–6.24) | 0.004 |
| Stage 3 vs Stage 2 | 0.87 (0.56–1.35) | 0.523 |
| Stage 4 vs Stage 2 | 1.40 (0.91–2.15) | 0.121 |
| Stage 4 vs Stage 3 | 1.62 (1.11–2.37) | 0.013 |

**S1B. AFS**

| **Comparison** | **Adjusted HR (95% CI)** | ***P* value** |
| --- | --- | --- |
| Stage 2 vs Stage 1 | 2.99 (1.03–8.69) | 0.045 |
| Stage 3 vs Stage 1 | 2.79 (0.99–7.90) | 0.053 |
| Stage 4 vs Stage 1 | 5.42 (1.95–15.07) | 0.001 |
| Stage 3 vs Stage 2 | 0.93 (0.56–1.57) | 0.798 |
| Stage 4 vs Stage 2 | 1.82 (1.11–2.96) | 0.017 |
| Stage 4 vs Stage 3 | 1.94 (1.27–2.97) | 0.002 |

**S1C. All-cause mortality**

| **Comparison** | **Adjusted HR (95% CI)** | ***P* value** |
| --- | --- | --- |
| Stage 2 vs Stage 1 | 2.08 (0.69–6.27) | 0.194 |
| Stage 3 vs Stage 1 | 2.23 (0.78–6.40) | 0.137 |
| Stage 4 vs Stage 1 | 4.17 (1.48–11.79) | 0.007 |
| Stage 3 vs Stage 2 | 1.07 (0.59–1.95) | 0.821 |
| Stage 4 vs Stage 2 | 2.01 (1.13–3.58) | 0.018 |
| Stage 4 vs Stage 3 | 1.87 (1.16–3.02) | 0.010 |

**S1D. Major amputation (Firth penalized Cox regression)**

| **Comparison** | **Adjusted HR (95% CI)** | ***P* value** |
| --- | --- | --- |
| Stage 2 vs Stage 1 | 8.70 (1.04–1133.83) | 0.045 |
| Stage 3 vs Stage 1 | 5.09 (0.60–664.18) | 0.161 |
| Stage 4 vs Stage 1 | 12.61 (1.66–1616.40) | 0.008 |
| Stage 3 vs Stage 2 | 0.59 (0.21–1.66) | 0.306 |
| Stage 4 vs Stage 2 | 1.45 (0.62–3.71) | 0.400 |
| Stage 4 vs Stage 3 | 2.48 (1.05–6.39) | 0.039 |

**S1E. CD-TLR**

| **Comparison** | **Adjusted HR (95% CI)** | ***P* value** |
| --- | --- | --- |
| Stage 2 vs Stage 1 | 1.74 (0.56–5.41) | 0.342 |
| Stage 3 vs Stage 1 | 1.37 (0.45–4.21) | 0.582 |
| Stage 4 vs Stage 1 | 1.03 (0.32–3.29) | 0.963 |
| Stage 3 vs Stage 2 | 0.79 (0.38–1.64) | 0.528 |
| Stage 4 vs Stage 2 | 0.59 (0.26–1.34) | 0.207 |
| Stage 4 vs Stage 3 | 0.75 (0.34–1.64) | 0.471 |

**Abbreviations:** AFS, amputation-free survival; BMI, body mass index; CD-TLR, clinically driven target-limb reintervention; CI, confidence interval; DCB, drug-coated balloon; HR, hazard ratio; MAE, major adverse events; WIfI, Wound, Ischemia, and foot Infection.

**Notes:** Values are adjusted hazard ratios with 95% confidence intervals from pairwise comparisons between WIfI stages. Models were adjusted for age, body mass index, hypertension, hyperlipidemia, atrial fibrillation, chronic kidney disease, preprocedural infrapopliteal runoff score, preprocedural pedal runoff score, calcification score, and infrapopliteal drug-coated balloon use. Pairwise comparisons were obtained by refitting the adjusted model with alternative reference categories; reported P values are nominal and were not adjusted for multiple comparisons. Age, BMI, and the three anatomical scores were modeled as continuous variables; binary covariates used absence or no use as the reference. Because no major amputation events occurred in stage 1, major amputation was analyzed using Firth penalized Cox regression with profile-likelihood confidence intervals and P values. All other outcomes used standard Cox proportional hazards regression.

**Supplementary Table S2.** Preprocedural angiographic characteristics by WIfI stage

| **Characteristic** | **Overall (n = 730)** | **Stage 1 (n = 91)** | **Stage 2 (n = 160)** | **Stage 3 (n = 257)** | **Stage 4 (n = 222)** |
| --- | --- | --- | --- | --- | --- |
| Preprocedural infrapopliteal runoff score | 10.12 ± 3.88 | 8.24 ± 3.43 | 10.19 ± 3.81 | 10.84 ± 3.87 | 9.99 ± 3.86 |
| Preprocedural pedal runoff score | 2.13 ± 0.70 | 1.99 ± 0.80 | 2.08 ± 0.70 | 2.20 ± 0.62 | 2.13 ± 0.73 |
| Number of diseased infrapopliteal arteries |  |  |  |  |  |
| 1 | 59 (8.1) | 14 (15.4) | 12 (7.5) | 14 (5.4) | 19 (8.6) |
| 2 | 188 (25.8) | 37 (40.7) | 49 (30.6) | 54 (21.0) | 48 (21.6) |
| 3 | 483 (66.2) | 40 (44.0) | 99 (61.9) | 189 (73.5) | 155 (69.8) |

**Notes:** Values are mean ± standard deviation or n (%). The unit of analysis is the study limb. Mean runoff scores and the prevalence of three-vessel infrapopliteal disease did not increase monotonically across WIfI stages.

**References**

1.	Mills JL, Conte MS, Armstrong DG, Pomposelli FB, Schanzer A, Sidawy AN, Andros G, Society for Vascular Surgery Lower Extremity Guidelines Committee. The Society for Vascular Surgery Lower Extremity Threatened Limb Classification System: risk stratification based on wound, ischemia, and foot infection (WIfI). *J Vasc Surg* (2014) 59:220-234.e1–2. doi: 10.1016/j.jvs.2013.08.003
